# The Immunosuppressive Activity of Heat Shock Protein 70

**DOI:** 10.1155/2012/617213

**Published:** 2012-12-17

**Authors:** Pawel Stocki, Anne M. Dickinson

**Affiliations:** ^1^Haematological Sciences, Institute of Cellular Medicine, Newcastle University, Newcastle upon Tyne NE2 4HH, UK; ^2^Department of Biochemistry, Medical Sciences Building, University of Toronto, 1 King's College Circle, Toronto, ON, Canada M5S 1A8

## Abstract

Heat shock protein 70 (HSP70) has previously been described as a potent antitumour vaccine. The mechanism relied on the ability of tumour derived HSP70 to associate with antigenic peptides, which, when cross presented, elicited a T cell mediated antitumour response. Subsequently, HSP70 was incorrectly described as a potent adjuvant of innate immunity, and although mistakes in the experimental approaches were exposed and associated with endotoxin contamination in the recombinant HSP70 specimen, questions still remain regarding this matter. Here we review only publications that have cautiously addressed the endotoxin contamination problem in HSP70 in order to reveal the real immunological function of the protein. Accordingly, “endotoxin free” HSP70 stimulates macrophages and delivers antigenic peptides to APCs, which effectively prime T cells mediating an antitumour reaction. Conversely, HSP70 has potent anti-inflammatory functions as follows: regulating T cell responses, reducing stimulatory capacity of DCs, and inducing development of immunosuppressive regulatory T cells. These activities were further associated with the immune evasive mechanism of tumours and implicated in the modulation of immune reactivity in autoimmune diseases and transplant-related clinical conditions. Consequently, the role of HSP70 in immune regulation is newly emerging and contrary to what was previously anticipated.

## 1. Tumour-Specific Antigens and Heat Shock Proteins

Numerous recent advances in the field of HSP-mediated immune regulation were inspired by the work of Pramod Srivastava. Srivastava advanced initial observations from the field of tumour immunisation and contributed greatly to the development of HSP-mediated tumour-specific immunotherapy. However, original research dates as far back as 1905 when Clowes and Baeslack observed that serum from a mouse, which had spontaneously recovered from a transplanted tumour, could give rise to antitumour immunity when injected into another inbred mouse with the same tumour [1]. Since then scientists have been searching for “the immune substance,” as a potential antitumour therapy. In 1943 Gross investigated immune resistance against tumours using the methylcholanthrene induced sarcoma (MC-Sa) mouse model, which he called “sarcoma 1” [2]. Gross intradermally injected a cell suspension of “sarcoma 1” into an inbred strain of mouse [2] and those mice, which spontaneously recovered from the transplanted tumour, gained resistance to subsequent attempts of tumour “sarcoma 1” induction [2]. However, the immunised mice were only resistant to “sarcoma 1” and were not resistant to spontaneous mammary tumours [2]. In 1957 Prehn and Main used a similar model to Gross [2] and generated a number of mice with MC-Sa sarcomas [3]. In a comparable set of experiments they showed that mice, which had spontaneously recovered from a tumour, gained resistance to the same MC-Sa tumour, but not to other MC-Sa tumours [3]. Some years later, in 1960, Klein et al. reported that irradiated tumour cells could be used to induce immunity but only to the same tumour from which the cells were taken [4]. In addition, the same authors showed that the immunisation was also effective when used in unrelated mouse lines [4].

In the following years research focused on the identification of “the immune substance,” which is currently referred to as tumour-specific antigens (TSAs). In 1982 DuBois et al. isolated TSA, which when used for immunisation, induced resistance to subsequent tumour transplantation. This protein of 75 kDa was purified from two distinct mouse MC-Sas, Meth A and CI-4 [5]. Antibodies raised against the 75 kDa TSA purified from Meth A were shown to also recognise the 75 kDa TSA from CI-4 [5]. Interestingly, the 75 kDa TSA isolated from Meth A induced protection against the Meth A tumour but not against CI-4 or other tumours [5]. In the same way the 75 kDa TSA purified from CI-4 induced resistance only to the CI-4 tumour [5]. Although it has never been confirmed, the size of the protein and its characteristics indicate that the 75 kDa TSA was in fact HSP70. Later, Srivastava et al. identified a glycoprotein of 96 kDa (gp96) as another TSA [6]. After it became apparent that gp96 is a very conserved protein, and could not induce a tumour-specific response by itself, the idea that gp96 works as an antigen carrier emerged [7]. A few years later, in a similar set of experiments, HSP70 was shown to give rise to antitumour immunity and that activity was solely dependent on the associated peptides and not the protein itself [8]. The process, from which the antitumour response was developed by HSP immunisation, was further attributed to cross presentation [9]. Accordingly HSPs, which induce such a response, had to be bound to MHC epitopes or epitope precursors upon purification. Indeed, our analysis of HSP70 associated peptides revealed the presence of MHC class I and class II epitope precursors bound to purified HSP70 [10, 11]. In accordance with our observation that HSP70 associates not only with MHC class I but also with class II epitopes, were previous observations that showed the importance of CD8^+^ and CD4^+^ cells in HSP70 mediated antitumour response [12].

## 2. Tantrum about Proinflammatory Functions of HSP70

By the early 2000s the mechanism by which tumour purified HSP70 with associated peptides can elicit an antitumour response was relatively well understood [13]. However around this time a very controversial article about the “danger signal” properties of HSP70 was published in a very prominent journal [14]. The phenomenon was very attractive because it packaged two important properties of an antitumour vaccine: antigen carrier and immune adjuvant, in one-HSP70. Many additional publications reporting on this mechanism appeared shortly after the initial paper. Unfortunately they overlooked that the a recombinant specimen of HSP70 (same HSP70 specimen that was used by Asea et al.—from StressGen, now Enzo) only induced an immune response when endotoxin levels were high and was completely abrogated after removal of the contamination [15, 16]. Disregarding this fact, the proinflammatory functions of HSP70 were still promoted in many reviews [17–21]. Since then the situation has not much changed and even the most recent reviews by prominent scientists describe unjustifiably all HSPs, including HSP70, as damage-associated molecular patterns (DAMPs) that facilitate proinflammatory responses following tissue injury or cell death [22, 23]. Controversy surrounds many HSPs and their proinflammatory roles, and mainly results from endotoxin contamination artefacts which seems to be commonly overlooked due to a dogmatic approach adopted by some of the scientific community. Many reviews were written and argued for or against mammalian HSP70 proinflammatory functions; however the fact remains that in those publications where the striking “danger signal-like” proinflammatory activity of HSP70 was shown, researchers used the endotoxin contaminated recombinant protein provided by Stressgen Biotechnologies [16, 25, 26]. Therefore in this paper we dismiss any data from studies where HSP70 endotoxin contamination was neglected and we will concentrate only on reports where HSP70 purified from a nonbacterial origin was used or studies where endotoxin contamination was stringently controlled.

## 3. Occam's Razor-Endotoxin-Free HSP70 and APC Modulation

So when one excludes those reports investigating the role of HSP70 using the recombinant specimen, whilst neglecting the possibility of endotoxin contamination, what are they left with? In fact there are quite a number of publications which may reveal the true function of HSP70. In the studies where HSPs were purified from a nonbacterial origin it was shown that HSP70 could stimulate macrophages to secrete cytokines and induce maturation of DCs [27]. However, one has to be aware that the HSP70 mediated DC activation profile was not only minor but also partial, and different from that influenced by LPS or gp96 [27]. In addition, the experiment indicating HSP70 induction of DC maturation was *n* = 1, which makes it impossible to interpret and questions reproducibility [27]. Later on, the same group tested the responses of primary murine macrophages as well as human myeloid cell lines and were able to show that exposure to HSP70 or gp96 can elevate nitric oxide secretion [28]. Of note, the experiments regarding DC maturation were only shown for gp96 and not for HSP70, which was possibly due to a lack of such activity [28]. Most recently it was shown that a 20 hour exposure of mouse macrophages to HSP70 at a concentration of approximately 100 *μ*g/mL, induced secretion of a unique selection of cytokines, mainly IL-1*β* and IL-6 but not TNF*α* [29]. Accordingly these experiments have shown that HSP70 does have some influence on immune response; however the patterns did not resemble those typical of DAMPs or endotoxins, and these responses seemed to mainly come from macrophages and not other antigen presenting cells such as DCs. Accordingly, the lack of DC maturation upon exposure to HSP70 was reported by others using concentrations as high as 200 *μ*g/mL [30]. Regarding DC maturation as an innate immunity activation measure there are also three additional publications that used “endotoxin free” recombinant HSP70 and showed no stimulatory activity towards monocyte derived DCs [15, 31, 32]. Moreover, it was also observed that the lack of this adjuvant property of HSP70 did not affect its ability to deliver antigenic peptides for effective cross presentation to T cells [15, 31, 32]. We also tested the proinflammatory capacity of HSP70 purified from two tumour cell lines, K562 and CCRF-CEM, on human monocyte derived immature DCs (mo-iDCs). Using a range of concentrations up to 10 *μ*g/mL we were unable to observe any stimulation of mo-iDCs maturation unlike with recombinant HSP70 (unpublished data). We also used a HSP70 specimen provided by Britta Eiz-Vesper and again failed to observe any significant expression changes in the tested DC maturation markers, that is CD83, CD80, CD86, or MHC class II at a concentration of up to 80 *μ*g/mL of HSP70 [26]. However we observed that a 24 hour exposure of mo-iDCs to HSP70 was able to visibly change their cluster formation pattern, which we commonly associate with the maturation status of mo-DCs [26]. We also noticed that mo-iDCs, even when left in a media without any stimuli, would still spontaneously mature as measured by CD83, CD80, CD86, or MHC class II expression as well as cluster formation ability (unpublished data). We therefore speculated that HSP70 could delay this spontaneous process rather than actively reprogramming the cells resulting in a reduced stimulatory capacity of mo-iDCs towards T cells [26]. It is also important to note that these observations were only consistently reproducible when serum-free medium was used and further work is needed to draw a conclusion from these data.

In conclusion, it can be summarised that HSP70, in high concentrations, can moderately stimulate a unique pattern of responses in macrophages but HSP70 fails to activate DC maturation. In contrast, even at low concentrations, HSP70 can reduce the stimulatory capacity of DCs [26]. Consequently, it has to be clearly stated that HSP70 does not qualify as a DAMP [33].

## 4. Endotoxin-Free HSP70 and T Cell Modulation

Recently HSP70 was also shown to improve survival of a neuroblastoma cell line upon heat stress and stimulated intracellular calcium flux in a monocytic leukaemia cell line [34]. It was also able to moderately induce cytokine secretion of mouse splenocytes and selectively changed their profile by increasing representation of CD4^+^ and CD11^+^, but not CD8^+^, by approximately 10–15% after 4 day incubation with 100–200 *μ*g/mL of HSP70 [34]. Also it was recently suggested that 7 day incubation of HSP70 at a concentration of 10 *μ*g/mL, can promote a unique T cell cytokine secretion profile; however the results are difficult to interpret since no statistical significance was reached [35]. One fact in agreement with the previous study by Zheng et al., was that proliferation of CD4^+^ cells upon the extended exposure to HSP70 was increased by approximately 15% (*P* < 0.01) followed by a 4% (*P* < 0.01) improvement of cytotoxic activity towards K562 and B-LCL [35]. No significant change in granzyme B secretion or production was found in any of the tested T cell subpopulations upon the extended HSP70 incubation. Of note, some of the anticipated HSP70 activities in T cell induction were found when additional cytokines were added alongside the protein, either IL-2 or a cocktail of IL-7, IL-12, and IL-15 [35]. However it has to be remembered that the addition of HSP70 to the media can improve cell viability in tissue culture conditions [26, 34]. Thus it can be speculated that changes in cell representation, proliferation, and cytokine secretion might have originated from the improved survival rather than the proinflammatory function, especially if extended exposure had taken place. In addition, the minor changes in certain experimental readouts in both of the studies reviewed above, although statistically significant, cannot be considered physiologically relevant without further studies. In our own study we used an HSP70 specimen provided by Britta Eiz-Vesper that was also used by Figueiredo et al. as described above. Surprisingly we found that use of the same protein sample, although in a different experimental setup, produced somewhat contradictory results. We examined both activated (whole CD3^+^/CD25^+^ population) and non-activated (CD3^+^/CD25^−^, CD4^+^/CD25^−^ and CD8^+^/CD25^−^ populations) T cell proliferative responses upon stimulation with HSP70-preincubated mo-iDCs. We observed that this pre-treated mo-iDCs had lower stimulatory capacity towards T cells [26]. However the additional supplementation of the media with HSP70 during the proliferation assay resulted in further reduction of T cell proliferation, indicating a direct effect of HSP70 on not only mo-iDCs but likely also on T cells. This was further confirmed when the activated T cell proliferation induced by IL-2 was reduced by HSP70. Also upon PHA stimulation the proliferative and secretory responses of activated and non-activated T cells (CD3^+^, CD4^+^ and CD8^+^) were significantly reduced by HSP70 [26].

In summary, the results obtained to date cannot be considered conclusive since even using the same specimen of HSP70 produced opposing results in differently arranged *in vitro* experiments. Again, our results showed a strong phonotype in T cell response reduction and these were common to all T cell subpopulations, while the opposing studies showed rather minor increases in T cell responses; however these were more specific to some T cell subpopulations in particular. Accordingly, in order to remain fair to currently available data, the role of HSP70 in influencing T cell responses requires independent verification. Importantly however, some relevant data supporting the reduction in T cell responses by HSP70 are coming from the studies examining the function of membrane bound HSP70 or using microbial HSP70 and are discussed below.

## 5. HSP70-Mediated Immunosuppressive Regulation

Our observations regarding the immunosuppressive activity of HSP70 were unexpected, given the common, yet incorrect association of HSPs with proinflammatory activity. However there is great body of supporting evidence coming from the field of microbial HSP70 where it has been used to treat different autoimmune diseases. It was shown that microbial HSP70 mediates anti-inflammatory reactions in different chronic inflammatory diseases models. The mechanism, although not entirely clear, points towards development of immune regulatory T cells (Tregs) reactive to self-HSPs and is mediated by an increase of IL-10 production [36]. Similar to our data showing the effect of human HSP70 on both DC maturation and T cell responses [26], microbial HSP70 was demonstrated to delay the DC maturation process and independently reduced proliferative response of T cells upon PHA stimulation [37, 38]. This immunosuppressive activity of microbial HSP70 was later tested in an animal allograft rejection experiment and showed that the skin or tumour allograft rejection can be delayed by preincubation with microbial HSP70. It was also shown that the microbial HSP70 mediated immunosuppressive and tolerising effect was abrogated upon Tregs depletion [39]. Moreover subcutaneous injection of 30 *μ*g of microbial HSP70 was shown to increase representation of Tregs in excised draining lymph nodes. In addition, the lymph node derived cells were shown to be less responsive to PHA stimulation and secreted approximately 25% more IL-10 and approximately 40% less TNF*α* [39]. These very interesting data correlate very well with our own observation, thus it would be of great interest to compare the immunosuppressive activity of microbial HSP70 with either human or endogenous murine HSP70. Such experiments would show if microbial HSP70 shares the same immunosuppressive capacity with endogenously expressed HSP70 or if it is a result of some other mechanism.

HSP70 upregulation, active secretion, and abundant cell membrane expression are common to tumour cells [40, 41]. Moreover, HSP70 expression is specific to tumour cells and has not been observed in normal cells. In many cases it was associated with poor survival possibly also resulting from poor responses to chemotherapy [42, 43]. Therefore HSP70 can be associated with the immune evasive mechanism of tumour cells. In fact it has recently been shown that HSP70 is indeed being utilised by tumour cells to promote immunosuppression. Tumour cells were found to produce exosomes, which were loaded with membrane bound HSP70. These if incubated with myeloid-derived suppressor cells (MDSCs) induced Stat3 phosphorylation and led to the development of immunosuppressive activity in MDSCs [44]. Knock-down of HSP70 in tumour cells was shown to abrogate the effect of exosomes on MDSCs and the same abolition was obtained when the exosomes were preincubated with anti-HSP70 neutralising antibodies [44]. The role of HSP70 in mediating immunosuppressive activity of MDSCs is very interesting, given that MDSCs are one of the major suppressors of antitumour activity that also reduces CD8^+^ T cell antigen recognition of tumour cells [45].

HSP70 upregulation was found in many immune mediated diseases. High expression of HSP70 was observed in human patients as well as animal and tissue models of graft versus host disease (GvHD), a major complication of haematopoietic stem cell transplantation. The underlying cause of the disease is an alloreaction of the engrafted immune cells against the host. Increased expression of HSP70 in rat spleen and lymph nodes corresponded with progressive GvHD [46]. Upregulation of HSP70 expression has been demonstrated in a skin explant model of GvHD and correlated with an increased grade of graft versus host reactivity in the biopsy [47]. Increased HSP70 expression has also been reported in the field of solid organ transplantation where rejection of rat cardiac allografts was accompanied by an upregulation of HSP70 by cardiomyocytes [48]. A strong upregulation of HSP70 has also been observed in the epithelial layers and mononuclear cells of colon biopsies from patients with inflammatory bowel diseases [49]. Furthermore HSP70 expression has been the subject of rheumatoid arthritis (RA) research and high expression of HSP70 has been reported in RA patients' synovial fluids [50]. All of these diseases were shown to be associated with HSP70 upregulation in the target rather that effector cells as a consequence rather than a cause of immune reactivity.

These data, in conjunction with the recently revealed immunosuppressive function of HSP70, can argue that upregulation of this protein in various clinical conditions is associated with the protective anti-inflammatory regulation that cells utilise upon a cytotoxic reaction of the immune system. However, this mechanism of cellular response, although feasible, will have to be further examined using a robust experimental approach.
